# A Selection in Cattle as a Preventive of Disease among People

**Published:** 1895-08

**Authors:** John Ernst


### A SELECTION IN CATTLE AS A PREVENTIVE OF DISEASE AMONG PEOPLE.[1]

BY JOHN ERNST, V.S.

In making a selection we do not intend to speak of a certain breed of a certain cattle, but to select such cattle, if possible, as possess no predisposition to disease, and attempt to breed with a view to preventing disease among the bovine race, and by so doing try and relieve our brethren from some of the diseases they suffer.

This should include those contagious diseases that afflict mankind when partaking of the flesh and milk of cattle so diseased.

Different forms of treatment have been tried. Medicine acts very well in some cases, whilst in others it does not. Of those that it does not we should try some other method that may in time prove to be of some benefit. It may be impossible to entirely prevent contagious diseases, but they may be prevented to a more or less extent by good hygiene and other measures that are natural to the animals. The subject of predisposition to disease and measures to escape it may lead to a beneficial result if properly and accurately studied and put into effect. Heretofore the aim in breeding good stock has consisted in color, form, and size, or the most pounds of beef or milk with the least amount of food in the shortest length of time. To prevent contagious diseases among cattle we should attempt to dispose of the predisposition to disease if such be possible.

[1] Read at the June meeting of the Missouri Valley Veterinary Medical Association.

To do so we must learn the cause, and by removing it improve the health of cattle and ourselves.

It has been said that the present system of breeding cattle, such as full-blood and high-grade, has a tendency to produce conditions predisposing to contagious diseases. If this be correct, and the cause removed, the conditions will correct themselves.

A rapid development or growth of tissue seems to be accompanied by a peculiarly delicate condition, let it be animal or vegetable. We see that most all vegetation of rapid growth soon dies and the tissues soon decay, while those that grow more slowly live longer, the tissues are more firm and decay less rapidly. If this be natural in cell-development, we may conclude that a rapid development of tissues has a tendency more or less to give rise to a condition favorable to degeneration of tissues and development of germs in cattle. Should this be true, the present system of raising beef does not comply with methods favorable to good health, as the forcing system and rapid development of the animal are the principal features of stock-raising.

Acknowledging the liability to disease among people from the use of milk and beef, we may say that by treating the cow we treat the human family.

That which constitutes hardiness, or a condition to withstand disease, is a condition that we know but little of, other than through methods of breeding. Perhaps the better way to obtain a knowledge of the cattle that are comparatively free is to keep a record of the herds and families that have been known not to have been affected to any extent for as many years as can be ascertained. If it be found at any time that one or more of a herd be affected, the breeding should be questioned and a careful study be made with a view to correct the weakness and tendency to disease.

The short-horn and Herefords, with the Jersey and Holstein, are the cattle that are the favorites in this country, or most frequently raised. As defects exist in all the different breeds as far as we know, the efforts of the breeder should be to unite such blood as will produce a condition unfavorable to the development of disease. The cows that show delicate constitution should not be chosen as the head of the herd, that is, thin skin that looks as if the blood could be squeezed through easily. The Jerseys afford a fair example for a poor choice, as they have thin lips, thin and delicate eyelids, ears light, legs and feet small and delicate looking, as if Nature intended they should not bear much hardships. Every organ should be proportioned in size and strength to other organs of the body. The general conformation should be consistent with form and durability so as to be able to withstand the demands of excessive functional activity if necessary. But the stock-raiser will object to this, as he will contend that he is not so well paid for his feed and labor in raising such cattle, as they will not suit the ideas of buyers, and he does not obtain the number of pounds of beef and milk that he would otherwise. If the public were aware that he bred his stock with a view to health as well as profit in dollars and cents they would prefer his to those of his neighbor who only expected remuneration in money.

The hardy-looking cattle do not always prove to be the least liable to hereditary diseases, thus we cannot always depend on appearances as to their having a tendency to be free of predisposition. Some method of keeping a record may accomplish this. If the breeding be properly managed it may produce immunity to disease. With a view to health as the first principle of breeding, we trust that humanity will meet with profit by evading human suffering.

